# An Open Letter to Whom It May Concern

**Published:** 1892-01

**Authors:** 

**Affiliations:** Kingston, Jamaica


### AN OPEN LETTER TO WHOM IT MAY CONCERN.

By the Monocrat of the Dinner-Table.

Many persons, apparently intelligent, and even many homœopathic physicians of claimed intelligence and known ability, have a brain so constituted that they cannot accept the idea of the efficacy and curative range of high potencies, or even that such potencies ever have any effect either for good or ill upon the human system or any other animal economy. With such knowledge of many of my *confrères* and of the world at large, I cannot refrain from asking them to explain the following statement, which is a provable verity:

I live in a tropical country that is infested with ants of all sizes, colors, and descriptions. Some of them are very destructive, mastering everything except iron, steel, and two or three varieties of hard wood—even killing full-grown trees.

Others are an intolerable nuisance in the house, such as the extremely minute "sugar" ant, the small red ant, and the medium-sized, but war-like, black ant.

Four years ago I moved into my present abode, and upon a two and a half by four feet varnished mahogany table I placed my fifteen by twenty-inch black walnut case, holding one hundred and fifty half-ounce vials of homœopathic medicines, the majority of which contained thirtieth potencies in the form of pellets, small, number eight. For very many weeks I was greatly annoyed by the "sugar" ants and the red ants, which literally swarmed among my medicines. After a time they all disappeared. In about eight or nine months I had occasion to prescribe my Berberis-vulgaris, when to my amazement the bottle was *totally empty*, whereas when I placed the case upon the table that bottle was seven-eighths full of number eight pellets of Berberis-vulgaris in thirtieth potency. Examination revealed that the ants had found a small hole in the side of the cork in which they burrowed until they came against the neck of the bottle, thence downward along the side of the cork and into the vial. Through this avenue they had carried away every solitary pellet, but even then must have carried it in pieces. Then it was that I judged that the pains of Berberis, and not respect for my wishes, had sent them *en masse* to happy hunting grounds, where creeds and pathies are not known. But, like all great discoverers, I allowed my discovery to sleep, and noted it not.

In another room, at the other end of the house, in a deep window-sill stands an earthen water-jug, or *porron*. For four years, almost daily, the tea-pot spout of this jug would be alive with red ants, which would follow into the glass on pouring.

The surface of the water in the jug would also show numerous little islands of red ants, locked together and floating. And, if the glass which was used for drinking was placed back upon the tray, it would speedily be filled with myriads of "sugar" ants, which are so minute as to be almost invisible save on glass or on something white. I tried various expedients of soap and water, insect powder, naphthaline, and scalding with hot water, on the window-sill and on their run-ways, but to no purpose, when finally, several weeks ago,

> A little bird whispered to me!
> And said, "Young man, use the B-V.!"

I quickly took the hint, put half a teaspoonful of my new stock of pellets of Berberis-vulgaris thirtieth into a small glass dish, moistened with water and placed in the window-sill near the water-jug. The "sugar" and red ants were delighted, and I looked on for nearly three days with much scientific interest. Then the ants lost HEART, I lost interest, and there was no longer anything to look at save the empty glass dish and the now respected water-jug, for the ants ceased to come, have not come since. I no longer drink ants, and for nearly four years they have not come on my medicine table. WHY?

KINGSTON, JAMAICA, September 10th, 1891.

